# Supplementary material for: Association between oral microbiome and seven types of cancers in East Asian population: a two-sample Mendelian randomization analysis
Source: Front Mol Biosci. 2023 Nov 21;10:1327893. doi: 10.3389/fmolb.2023.1327893 (PMC10702768; doi:10.3389/fmolb.2023.1327893)
Supplement: Supplementary file 1 [file DataSheet1.ZIP › Supplementary Materials/MR plots of saliva/saliva═╝/colorectal cancer/pheno.2288_to_colorectal cancer_leave_one_out.pdf]

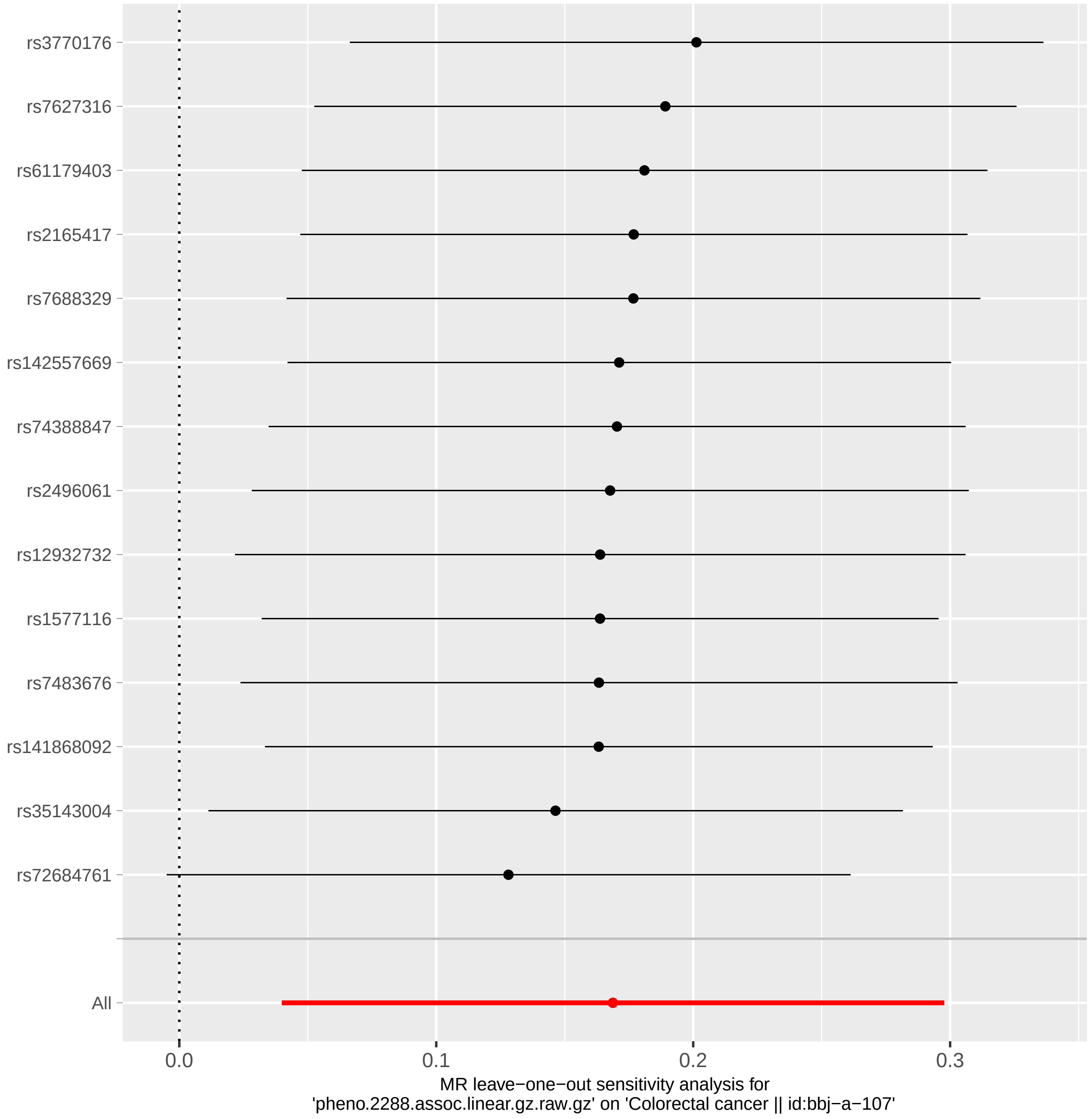

rs3770176
rs7627316
rs61179403
rs2165417
rs7688329
rs142557669
rs74388847
rs2496061
rs12932732
rs1577116
rs7483676
rs141868092
rs35143004
rs72684761
All
0.0
0.1
0.2
0.3
MR leave−one−out sensitivity analysis for
'pheno.2288.assoc.linear.gz.raw.gz' on 'Colorectal cancer || id:bbj−a−107'
